# Safety of Fixed Dose of Antihypertensive Drug Combinations Compared to (Single Pill) Free-Combinations: A Nested Matched Case–Control Analysis

**DOI:** 10.1097/MD.0000000000002229

**Published:** 2015-12-11

**Authors:** Emmanuel Nowak, André Happe, Jacques Bouget, Francois Paillard, Cécile Vigneau, Pierre-Yves Scarabin, Emmanuel Oger

**Affiliations:** From the Pharmacoepidemiology Team (CTAD-PEPI), Rennes, France (EN, AH, EO); Université Européenne de Bretagne, Université Europeenne de Bretagne, Université de Brest, INSERM CIC 1412, IFR 148 et CHU de Brest, France (EN); CHU de Brest, France (AH); Emergency Department (JB), Cardiology Department (FP), and Nephrology Department (CV), Rennes University Hospital, Rennes, France; ESH Hypertension Excellence Centre (FP, CV); INSERM, CESP, UMR-1018, Villejuif, Paris, France (P-YS), and Pharmacovigilance, Pharmacoepidemiology and Drug Information Center, Rennes, France (EO).

## Abstract

To compare serious adverse events of fixed-dose dual antihypertensive drug combination (FIXED) to component-based free-combination (FREE).

A population-based nationwide cohort from the French Health Insurance System included subjects over 50 years with first time claims (new user) in the second half of 2009 for a calcium-channel blocker or a thiazide-like diuretic in combination with either an angiotensin-converting enzyme inhibitor or an angiotensin receptor blocker as FREE or FIXED. We designed a nested matched case–control analysis with 304 cases, hospitalized for hypotension, syncope, or collapse (n = 224), renal failure (n = 19), hyponatremia, hyper- or hypokalemia (n = 61) and 1394 controls matched for gender, age, date of inclusion in the cohort, and administrative county. Subjects with a medical history of cardiovascular disease, chronic renal failure, or cancer were excluded.

The mean age ± SD was 73 ± 10 years and 70% were women. Based on the last delivery preceding the index date, 1414 patients (83%) were exposed to FIXED. Homogeneity of FIXED effect compared to FREE across components of the main composite outcome was rejected (*P* = 0.0099). FIXED formulation significantly increased the odd of the most frequent component (ie, hypotension, syncope, or collapse): OR = 1.88 (95% CI: 1.15–3.05) compared to FREE after adjusting for confounding factors including dose.

Serious adverse event occurring in the early phase of treatment deserves attention of physicians because it could alter the benefit/risk ratio of antihypertensive drug combination.

## Abstract 2

Supplemental Digital Content is available in the text

## INTRODUCTION

High blood pressure (hypertension) is 1 of the most important preventable causes of premature morbidity and mortality. The clinical management of hypertension is 1 of the most common interventions in primary care, accounting for at least a billion in drug costs alone in Western countries. The most recommended combinations of drugs are those associating an angiotensin-converting enzyme (ACE) inhibitor or an angiotensin receptor blocker (ARB) with either a calcium-channel blocker (CCB) or a thiazide-like diuretic (TZD).^1,2^ When more than 1 drug is prescribed, the use of a combination product with 2 appropriate medications in a single tablet (fixed-dose combination, FIXED) can simplify treatment for patients, though these products can sometimes be more expensive than individual drugs.^2^

One of the arguments put forward in promoting FIXED is better compliance.^3,4^ Meta-analyses have reported improved adherence and lower healthcare costs associated with FIXED compared with component-based combinations (FREE) of the same classes in treating patients with hypertension.^5–7^ However, data on the impact of FIXED in terms of reducing cardiovascular morbidity and mortality are sparse. Regarding side effects, a meta-analysis of Gupta et al^6^ was not conclusive, pooling existing data from 5 trials^8–12^ with small or medium sizes over a period ≤6 months and did not report information on the nature of side effects.

Our objective was to assess the risk of a serious adverse event of FIXED compared with FREE dual combination of the same active components in hypertensive patients.

## METHODS

### Data Source

We used data from the French National Health Insurance System, namely the health reimbursement database (Système National d’Information Inter-Régimes de l’Assurance Maladie, SNIIRAM) linked by a personal health unique number to the French hospital discharge database (Programme de Médicalisation des Systèmes d’Information; PMSI). We used data covering the period 2009 to 2011.

The Institutional Review Board of French Health Insurance System (Institut des Données de Santé) approved this study (no. 28, September 2011), as well as the Commission Nationale de l’Informatique et des Libertés (AE-111134).

### Study Design and Study Population

This is an observational population-based nationwide cohort study targeting hypertensive subjects over 50 years (born before December 31, 1958), initiating FREE or FIXED dual antihypertensive combination between July 2009 and December 2009 with a CCB or a TZD in combination with either an ACE inhibitor or an ARB. Initiation was defined by an absence of refund of FREE or FIXED for at least 6 months (first semester 2009). We designed a nested matched case–control analysis.

### Case Definition

Cases were subjects who were hospitalized after their inclusion in the cohort, for 1 of the following conditions: hypotension, syncope or collapse, acute renal failure, and hyponatremia, hyper- or hypokalemia. We used ICD-10 codes in primary hospital discharge diagnosis position (see Text document, Supplemental Digital Content 1). The date of hospital admission defined the index case date. We then defined 3 types of cases, thus concatenating some items: hypotension, syncope or collapse, acute renal failure, and hypo- or hyperkalemia or hyponatremia.

### Control Definition

Controls were subjects who did not correspond to the definition of a case at any time before the index date. Controls were matched for gender and age (±2 years). To ensure that cases were comparable to controls regarding the time between initiation of FREE or FIXED and the index date in case, matching was performed on the date of inclusion in the cohort within 28 days. Furthermore, in order to control for the role of climatic conditions on the occurrence of an event of interest (through dehydration in a context of heat), matching was also made on the area (administrative county) where subjects were living on the index date. The area was determined by hospital location for cases and by the most frequently recorded information for controls among all health spending reimbursements.

### Exclusion Criteria

Whatever the status, case or control, subjects having at least 1 of the following features at the index date (identified through hospitalization within the previous 6 months or long-term disease [LTD] status) were excluded from all analyses: Medical history of cardiovascular disease, hepatic insufficiency, systemic disease, chronic inflammatory arthritis, inflammatory bowel disease, organ transplantation, dementia, psychiatric disease, chronic renal failure, or cancer (see Text document, Supplemental Digital Content 1 and 2, which provides comprehensive LTD items and ICD-10 codes).

### Exposure Definition

Exposure of interest was a CCB or a TZD in combination with either an ACE inhibitor or an ARB as FREE or FIXED. Exposure at the index date was determined from deliveries related to the last antihypertensive drug prescription: subjects were considered to be exposed to FIXED if a delivery of a box of 28 or 30 tablets was recorded in 40 days before the index date (120 days for a box of 90 tablets); and no other drug belonging to the 4 therapeutic classes of interest has been supplied. Subjects were deemed exposed to FREE if a delivery was recorded for the 2 corresponding drugs each using the above-mentioned delay; and no other drug belonging to the 4 therapeutic classes of interest has been supplied. All other situations led to exclude the corresponding patient from analysis.

For a fair comparison (including a dose-adjusted 1), we considered for FREE combination only those components which were available in fixed-dose combination. The daily dose of each component was divided into 2 categories (low or medium vs high; see Table 1, Supplemental Digital Content 3 for list of components and medium dose).

**TABLE 1:**
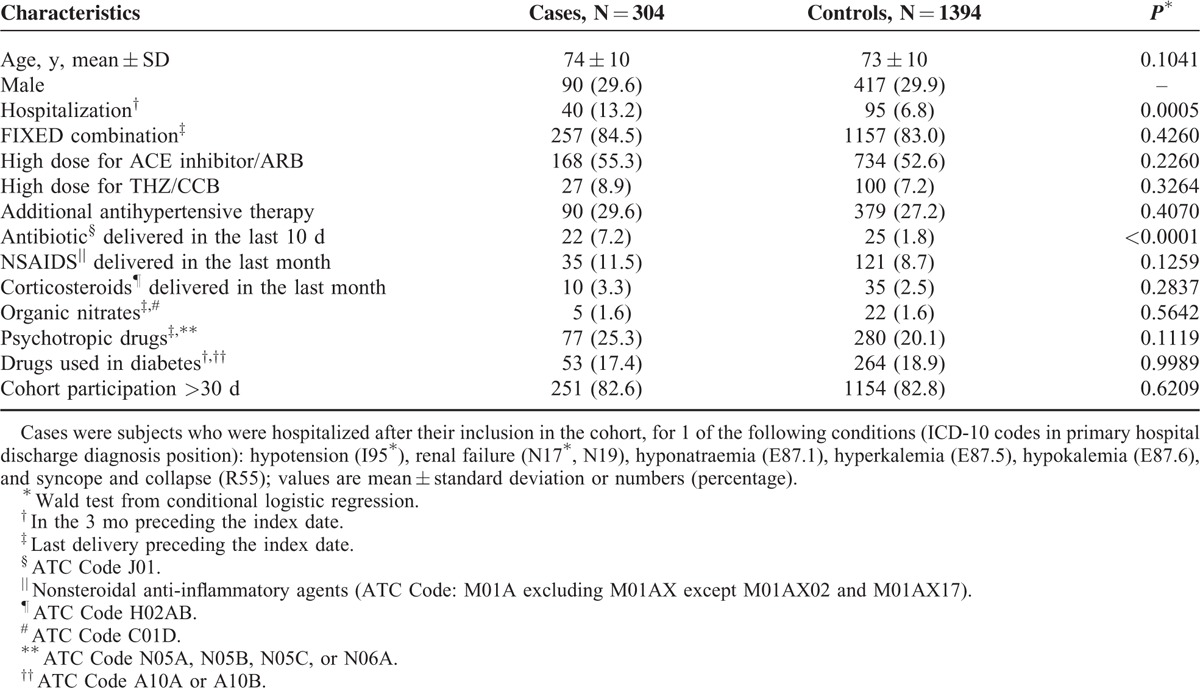
Characteristics of Cases and Matched Controls at the Index Date

### Covariates

Any hospitalization (ICD-10 codes) in the 3 months preceding the index date as well as last delivery of drugs (ATC code) before the index date, focusing on other antihypertensive agents, were also used as an attempt to control for confounding (see Text document, Supplemental Digital Content 2). We assessed the nature of antihypertensive therapy prior to initiation of the dual combination therapy. We also considered medical care in the 6 months preceding enrolment.

### Statistical Analysis

We first compared the characteristics of cases and their matched controls at the index date and according to FREE or FIXED at baseline (inclusion in the cohort).

The association between hospitalization for a serious adverse event and exposure was estimated by an odds ratio (and its confidence interval 95%) through conditional logistic regression. Other covariates were included in a multivariate model provided that they were associated at a 0.15 statistical level when cases were compared with matched controls. Dose level of targeted antihypertensive drugs was then forced in a final model. Homogeneity of associations across the individual components of the composite outcome was assessed by using an interaction term between exposure and individual outcomes in the conditional logistic model, according to Pogue et al.^13^

Lastly, a sensitivity analysis excluded all controls having had hospitalized any time during follow-up (from inclusion to index date, in order to exclude potentially false negative controls.

## RESULTS

We identified 304 cases and 1394 controls. The characteristics of these subjects are described in Table 1 (see Supplemental Digital Content 4 for characteristics at inclusion in the cohort).

The median (Q1–Q3) of occurrence of a qualifying event (composite outcome) was 182 days (67–395); these 304 events were acute renal failure, n = 19 (6%); hyponatremia, hypo- or hyperkalemia, n = 61 (20%); and hypotension, syncope, or collapse, n = 224 (74%).

Crude (with matching) and adjusted measures of association are shown in Table 2. Homogeneity of associations across individual components of the main composite outcome was rejected (*P* = 0.0099 for adjusted analysis including dose). Estimates were quite imprecise for “acute renal failure” and “hypo/hyperkalemia or hyponatremia.” Conversely, FIXED formulation significantly increased the odd of the most frequent component (ie, hypotension, syncope, or collapse): OR = 1.88 (95% CI: 1.15–3.05) compared with FREE after adjusting for confounding factors including dose.

**TABLE 2:**
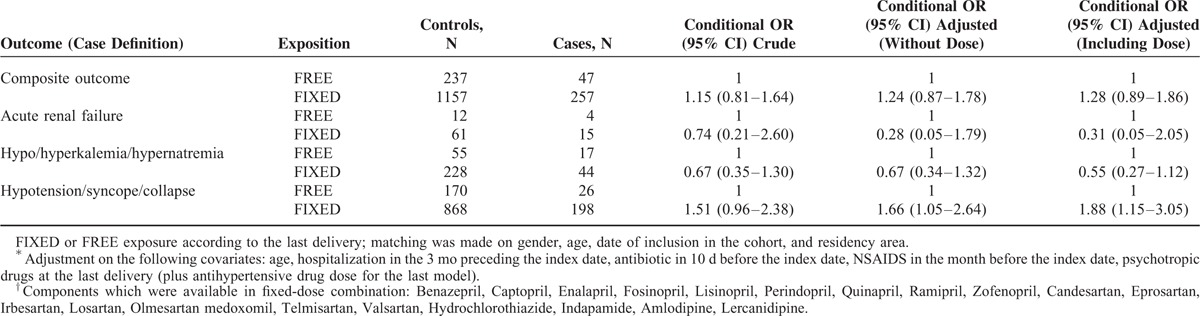
Crude and Adjusted^∗^ Matched Odds Ratios (OR) of Serious Adverse Event Hospitalization Among Patients Exposed to Fixed-Dose Combination of Antihypertensive Drugs Compared With Those Exposed to Single Pill Combination of the Same Active Components With No Additional Antihypertensive Drug of Interest Considering for Free Combination Only Those Drug Components Which Were Available in Fixed-Dose Combination^†^

Dose pattern (low-medium vs high) according to FIXED or FREE formulation for those 1698 patients (304 cases) were the following: THZ/CCB dose patterns (low-medium vs high) were 95% versus 5% for FIXED and 80% versus 20% for FREE formulation; ACE inhibitor/ARB dose patterns (low-medium vs high) were 47% versus 53% for FIXED and 49% versus 51% for FREE formulation. As anticipated, our data were consistent with a dose–effect relationship for CCB/THZ when considering the main component, that is, hypotension, syncope, or collapse: OR = 1.75 (1.01–3.05) for high versus low-medium. This was not observed for the ACE inhibitor/ARB dose: OR = 0.97 (0.71–1.31). There was no statistically significant interaction between dose and type of exposure (FREE vs FIXED) for hypotension, syncope, or collapse: *P* = 0.78 and *P* = 0.30 when considering ACE inhibitor/ARB dose and THZ/CCB dose, respectively.

Excluding all potentially false negative controls, a multivariate sensitivity analysis (with regard to hypotension, syncope, or collapse) led to OR = 2.06 (1.23–3.44) after adjusting for confounding factors including dose.

## DISCUSSION

### Main Findings

We used a large claim database to estimate the measure of association between serious adverse event with hospitalization and exposure to fixed-dose combination (FIXED) of antihypertensive drugs compared with single pill combination of the same active components (FREE). Homogeneity of association across individual components of the main composite outcome was strongly rejected. FIXED formulation significantly increased the odd of the most frequent component (ie, hypotension, syncope, or collapse): OR = 1.88 (95% CI: 1.15–3.05) compared with FREE after adjusting for confounding factors including dose.

### Comparison With Other Studies

There are only a few published studies that have examined safety issues for combination of antihypertensive drugs with mixed findings. Adverse effects were reported in 5 trials including a total of 1775 hypertensive patients.^8–12^ Meta-analysis^6^ of these studies showed a nonsignificant decrease in adverse effects associated with the use of a FIXED as compared with a FREE combination (OR: 0.80 [95% CI: 0.58–1.11]). But the meta-analysis pooled all adverse effects, serious or not and did not described them. The most common drug-related adverse events were asthenia and metabolic disorders including hyperkalemia or hypokalemia. Only 3 out of those 5 trials involved drugs we were interested in.^10–12^

In a large population-based study,^14^ the concurrent use of diuretics and ACE inhibitors or ARBs along with NSAIDs was associated with an overall 31% higher risk of acute kidney injury, which is driven by a nearly 2-fold increased risk in the first 30 days of use. The authors did not stratify according to fixed-dose combination or single pill combination and dose of antihypertensive drugs were not considered.

The UMPIRE trial was an open-label, randomized, blinded end-point trial comparing a fixed combination of aspirin, statin, and 2 antihypertensive agents (10 mg lisinopril and either 50 mg atenolol or 12.5 mg hydrochlorothiazide) with usual care in patients with established cardiovascular disease or at risk for cardiovascular disease.^15^ After a median follow-up of 15 months, no significant differences were found between the groups in terms of serious adverse events.

Recent guidelines have recommended a combination of 2 drugs to be used as first-step treatment strategy in high-risk hypertensive individuals to achieve timely blood pressure control and avoid early events.^16^ In a subset of patient especially elderly patients this option may be associated with an excessive effect and an increased risk of adverse effects.

As regard hypotension, syncope, or collapse, all drug components (CCB/TZD as well as ACE inhibitor/ARB) have an obvious dose-dependent deleterious effect. Plausibility of an association when comparing FIXED to FREE could rely mainly on 2 reasons. Firstly, 1 of FIXED inconveniences is the relative limitation to increase the dose of 1 drug independently of the other; or adapt the dose of 1 drug to some circumstances (taper diuretic dose in case of heat or diarrhea). Secondly, misunderstanding of the various active components of these associations, as well as their doses, might lead to prescriptions potentially cause of serious adverse event. Indeed, as expected, NSAIDS deliveries in the last month were more frequent in cases than in controls. Of note, to be reimbursed, NSAIDS had to be prescribed by a physician. In addition, antipsychotics, anxiolytics, hypnotics, and sedatives as well as antidepressants deliveries at the last pharmacy contact were more frequent in cases than in controls.

### Strengths and Limitations

Potential limitations arise from using data from health insurance database. Though we applied exclusion criteria to focus on hypertensive patients in a primary prevention setting, we could not rule out that some patients with other indication were analyzed. Classification bias for outcome may have arisen as we used ICD-10 codes in primary hospital discharge diagnosis position for classifying hospitalizations. We postulated that potential misclassification was nondifferential. A sensitivity analysis excluding all controls having had hospitalized any time during follow-up showed similar results. Because of the observational nature of this study, residual confounding by indication and disease severity may be present. However, we specifically dealt with this possibility by comparing 2 modalities of dual antihypertensive therapy combining the same components, assuming that contra-indications and indications were shared. We reckon that some relevant differences arose between cases and controls, and further adjustment was performed. It is of note that this adjustment made little variation on association estimate.

The strength of our analyses is that, based on a comprehensive nationwide health insurance database, the inclusion of all eligible patients ensures external validity. Information on use of antihypertensive drugs from a prescription database is highly reliable.

### Implications for Practice and Conclusions

From a global public health perspective, do our findings matter if FIXED had demonstrated, through better adherence, a decreased risk of major cardiovascular events compared with FREE? Firstly, serious adverse event occurring in the early phase of treatment might impact adherence to FIXED: patients being reluctant to resume taking this medication, physician being uneasy to maintain FIXED. Secondly, the validity of the demonstration of a decreased risk of major cardiovascular events with FIXED is questionable.^17,18^ The reduction in hospitalization rates^16^ was significant in the nonadherent subgroup suggesting that any risk reduction, if true, was not fully related to adherence.

Until head-to-head randomized control trials evaluate the effect of FIXED regimens compared with FREE on major cardiovascular outcomes in treating hypertensive patients, epidemiological data are useful to assist medical decision specifically when assessing association with rare events such as severe adverse events.

## Supplementary Material

Supplemental Digital Content
