# Supplementary material for: Safety of Fixed Dose of Antihypertensive Drug Combinations Compared to (Single Pill) Free-Combinations: A Nested Matched Case–Control Analysis
Source: Medicine (Baltimore). 2015 Dec 11;94(49):e2229. doi: 10.1097/MD.0000000000002229 (PMC5008510; doi:10.1097/MD.0000000000002229)
Supplement: Supplemental Digital Content [file medi-94-e2229-s001.doc]

Supplemental Digital Content 1

We used ICD-10 codes in primary hospital discharge diagnosis position for classifying hospitalizations: hypotension (I95*), syncope and collapse (R55) renal failure (N17*, N19), hyponatremia (E87.1), hyperkalemia (E87.5), hypokalemia (E87.6).

In France, 29 long-term diseases (LTD) provide full reimbursement of care. For a patient to be attributed an LTD status, the physician in charge must fill in a detailed form that provides date and criteria of diagnosis; the file is then reviewed by medical authorities for approval and an ICD-10 code is then validated. We thus use ICD-10 codes (LTD or hospitalization primary diagnosis) for exclusion.

Patients with one of the following long-term diseases (LTD) were excluded: disabling stroke, medullar insufficiency or other chronic cytopenia, chronic arterial disease with ischemic events, complicated schistosomiasis, severe heart failure or serious arrhythmias or severe valvular heart disease or severe congenital heart disease, coronary artery disease, active chronic liver disease or cirrhosis, severe primary immunodeficiency requiring prolonged treatment or infection with human immunodeficiency virus (HIV), severe neurological or muscular diseases (including myopathy) or severe epilepsy, constitutional or acquired severe hemoglobinopathy or hemolysis, haemophilia or severe constitutional hemostasis disorders, severe chronic respiratory failure, Alzheimer's or other dementia syndrome, Parkinson disease, inherited metabolic diseases requiring prolonged treatment specialist, cystic fibrosis, severe chronic nephropathy or primitive nephrotic syndrome, paraplegia, vasculitis, systemic lupus erythematosus, systemic sclerosis, active rheumatoid arthritis, chronic psychiatric disorders, ulcerative colitis and Crohn's disease, multiple sclerosis, severe spondylitis, organ transplantation, active tuberculosis, leprosy, malignant tumor, lymphatic or hematopoietic malignant disease.

ICD-10 codes for patients with type 1 and type 2 diabetes used for exclusion: multiple complications (E107, E117, E147), renal complication (E102, E112, E142), comatose (E110), acidocetosis (E101, E111, E141), vascular complication (E135), unlabeled complication (E138), retinopathy (H350).

ICD-10 codes for patients with hypertension used for exclusion: hypertensive heart disease (I11), hypertensive nephropathy (I12), secondary hypertension (I15).

Supplemental Digital Content 2

ICD-10 codes in primary hospital discharge diagnosis position or drug delivery ATC code in the 6 months preceding enrolment used as proxy for risk-factors profile

Cardiovascular risk proxy: C10 Lipid modifying agents; B01AC Platelet aggregation inhibitors excl. heparin: B01AC04 (clopidogrel), B01AC05 (ticlopidine), B01AC06 (acetylsalicylic acid), B01AC07 (dipyridamol), B01AC22 (prasugrel), B01AC23 (cilostazol), B01AC24 (ticagrelor), B01AC30 (combinations), B01AC56 (acetylsalicylic acid and esomeprazole); C01DA Organic nitrates;

Diabetes: diabetes mellitus (E10-E14) or drugs used in diabetes (A10: A10A insulins and analogues; A10B blood glucose lowering drugs, excl. insulins);

Depression or psychosis: delivery of psychotropic agent: antidepressants (N06A), antipsychotics (N05A), anxiolytics (N05B), hypnotics and sedatives (N05C);

COPD: emphysema (J43) or other chronic obstructive pulmonary disease (J44).

ICD-10 codes in primary hospital discharge diagnosis position in the 6 months preceding enrolment used as exclusion criteria: dialysis (09 11M05V, 09 11M06V, 09 11M05W, 09 11M06W, 10 11M06V, 10 11M06W, 10 11K02V, 10 11K02W,10 28Z01Z,10 28Z02Z,10 28Z03Z,10 28Z04Z,10 28Z05Z,10 28Z06Z, 11 11M06x, 11 11K021,11 11K022,11 11K023,11 11K024,11 11K02J,11 28Z01Z, 11 28Z02Z,11 28Z03Z,11 28Z04Z), antineoplastic chemotherapy or radiotherapy (10 28Z07Z, 10 28Z08Z, 10 28Z09Z, 10 28Z10Z, 10 28Z11Z, 10 28Z12Z, 10 28Z13Z, 10 28Z14Z, 11 28Z07Z, 11 28Z10Z, 11 28Z11Z, 11 28Z14Z, 11 28Z16Z, 11 28Z18Z, 11 28Z19Z, 11 28Z20Z, 11 28Z21Z, 11 28Z22Z, 11 28Z23Z, 11 28Z24Z, 11 28Z25Z).

ATC codes used to identify drug classes and create covariates

A10 Drugs used in diabetes: A10A Insulins and analogues; A10B Blood glucose lowering drugs. excl. insulins

C01DA Organic nitrates

C02 Antihypertensives (C02A Antiadrenergic agents. centrally acting; C02B Antiadrenergic agents. ganglion-blocking; C02C Antiadrenergic agents. peripherally acting; C02D Arteriolar smooth muscle. agents acting on; C02K Other antihypertensives; C02L Antihypertensives and diuretics in combination

C02N Combinations of antihypertensives).

C03 Diuretics: this group comprises diuretics. plain and in combination with potassium or other agents. Vasopressin antagonists are also included in this group. Potassium-sparing agents are classified in C03D and C03E.

C04 Peripheral vasodilators

C07 Beta blocking agents

C08 Calcium channel blockers: the calcium channel blockers are classified according to selectivity of calcium channel activity and direct cardiac effects: C08C selective calcium channel blockers with mainly vascular effects; C08D selective calcium channel blockers with direct cardiac effects; C08E non-selective calcium channel blockers; C08G calcium channel blockers and diuretics.

C09 Agents acting on the renin-angiotensin system

C10 Lipid modifying agents

B01AC Platelet aggregation inhibitors excl. heparin: B01AC04 (clopidogrel). B01AC05 (ticlopidine). B01AC06 (acetylsalicylic acid). B01AC07 (dipyridamol). B01AC22 (prasugrel). B01AC23 (cilostazol). B01AC24 (ticagrelor). B01AC30 (combinations). B01AC56 (acetylsalicylic acid and esomeprazole)

N05 Psycholeptics: The group is divided into therapeutic subgroups: N05A Antipsychotics; N05B Anxiolytics; N05C Hypnotics and sedatives; N06A Antidepressants.

M01A Anti-inflammatory and anti-rheumatic products. non-steroids. including M01AX02 (niflumic acid) and M01AX17 (nimesulide) but none of the other drugs labeled M01AX.

Supplemental Digital Content 3

**Table 1** Definition of dose according to pre-specified drug components which were available in fixed-dose combination; Medium dose was based on defined daily dose (WHO definition); any delivery of a package with a lower or higher unit dose was then classified as a low or high dose, respectively.

| Angiotensin receptor blocker | Angiotensin-converting enzyme inhibitor | Calcium-channel blocker | Thiazide-like diuretic | Medium dose |
| --- | --- | --- | --- | --- |
| Eprosartan |  |  |  | 600 mg |
| Irbesartan |  |  |  | 150 mg |
| Valsartan |  |  |  | 80 mg |
| Losartan | Captopril |  |  | 50 mg |
| Telmisartan |  |  |  | 40 mg |
|  | Zofenopril |  |  | 30 mg |
|  |  |  | Hydrochlorothiazide | 25 mg |
| Olmesartan | Lisinopril |  |  | 20 mg |
|  | Fosinopril, quinapril |  |  | 15 mg |
|  | Enalapril | Lercanidipin |  | 10 mg |
| Candesartan |  |  |  | 8 mg |
|  | Benazepril |  |  | 7.5 mg |
|  |  | Amlodipin, |  | 5 mg |
|  | Perindopril |  |  | 4 mg |
|  | Ramipril |  |  | 2.5 mg |
|  |  |  | Indapamide | 1.25 mg |

Supplemental Digital Content 4

**Table 2** Baseline characteristics of patients according to the type of antihypertensive therapy they initiated: component-based (single pill) free-combination therapy or fixed-dose combination (two drugs in a single tablet)

| Characteristics | FREE  N = 291* | FIXED  N = 1407* | p ^0^ |
| --- | --- | --- | --- |
| Age, years mean ± standard deviation  min, Q1, Q3, max | 75 ± 10  51, 70, 83, 93 | 73 ± 10  50, 65, 80, 96 | 0.0002 |
| Male | 86 (29.5) | 421 (29.9) | 0.9005 |
| Cardiovascular-related hospitalization ^1. 2^ | 0 | 0 | - |
| Diabetes mellitus ^1. 3^ | 70 (24.0) | 241 (17.1) | 0.0054 |
| Depression or psychosis ^4. 5^ | 105 (36.1) | 408 (29.0) | <.0001 |
| Previous antihypertensive therapy  Single therapy with a class of interest  other  Dual therapy including a class of interest  other  Three drugs or more including a class of interest  other  None | 186 (63.9)  12 (4.1)  66 (22.7)  4 (1.4)  18 (6.2)  3 (1.0)  2 (0.7) | 901 (64.0)  67 (4.8)  293 (20.8)  8 (0.6)  95 (6.7)  22 (1.6)  21 (1.5) |  |
| Antihypertensive therapy at inclusion  Three drugs (dual therapy of interest plus)  A class of interest  Other  Four drugs or more (dual therapy of interest plus)  At least a class of interest  Other  Dual therapy of interest alone | 0 (0.0)  90 (30.9)  1 (0.3)  17 (5.8)  183 (62.9) | 11 (0.8)  242 (17.2)  10 (0.7)  43 (3.1)  1101 (78.2) |  |
| Platelet aggregation inhibitors ^4. 6^ | 74 (25.4) | 269 (19.1) | 0.0147 |
| Lipid modifying agents ^4. 7^ | 146 (50.2) | 696 (49.5) | 0.8267 |
| Organic nitrates ^4. 8^ | 19 (6.5) | 25 (1.8) | <.0001 |
| Hospitalization ^9^ | 42 (14.4) | 155 (11.0) | 0.0976 |
| More than one general practitioner consultation ^9^ | 275 (94.5) | 1300 (92.4) | 0.2070 |
| More than one cardiologists’ consultation ^9^ | 7 (2.4) | 49 (3.5) | 0.3490 |
| More than one nephrologists’ consultation ^9^ | 1 (0.3) | 1 (0.1) | 0.3135 |

* Values are mean ± standard deviation or numbers (percentage); ^0^ Student T test t, chi-square test or Fisher test; ^1^ Hospitalization in the 6 months preceding the first delivery of the dual therapy of interest; ^2^ Main diagnosis: cerebral infarction (I63); cardiomyopathy (I42) or heart failure (I50) or hypertensive heart disease with (congestive) heart failure (I11.0); chronic ischemic heart disease (I25); Peripheral vascular disease (I73.9);^3^ Main diagnosis of diabetes mellitus (E10-E14) or anti-diabetic drug delivery (ATC code: A10A or A10B); ^4^ At least one delivery in the 6 months preceding the first delivery of the dual therapy of interest; ^5^ Antidepressants (N06A) Antipsychotics (N05A) Anxiolytics (N05B); ^6^ ATC code: B01AC04-B01AC07 B01AC22-B01AC24 B01AC30 or B01AC56 ^7^ ATC code: C10 ^8^ ATC code: C01DA; ^9^ In the 6 months preceding the first delivery of the dual therapy of interest.
